# Reduction of healthcare access inequity using telehealth and patient travel cost subsidisation

**DOI:** 10.1016/j.puhip.2024.100542

**Published:** 2024-08-23

**Authors:** Edwin Phillip Greenup, Daniel Best

**Affiliations:** Clinical Excellence Queensland, Queensland Health, Brisbane, Australia

**Keywords:** Telehealth, Telemedicine, Patient travel, Rural and remote Care

## Abstract

**Objective:**

Telehealth and patient travel cost subsidisation are two strategies used to reduce the effects of healthcare access inequity. Despite this shared goal, these programs are usually run independently, and their effects are infrequently compared in evaluation. Understanding how these programs are used helps ensure services are delivered efficiently.

**Methods:**

Counts of telehealth outpatient service events (TH) (n = 250171) and patient travel subsidy scheme claims (PTSS) (n = 270933) for the 2022-23 financial year were captured. Comparisons of PTSS and TH activity were made by postcode, rurality (The Accessibility/Remoteness Index of Australia (ARIA)) and health jurisdiction (Hospital and Health Service (HHS)).

**Results:**

Correlation analysis conducted on PTSS and TH activity revealed a statistically significant, moderate positive correlation (r = 0.449, p < 0.01). TH (coefficient = 0.650, p < 0.001) and rurality (coefficient = 26.208, p = 0.686) also retained their significance.

**Conclusions:**

This study established that increases in TH activity is correlated with increases in PTSS, with both programs reporting greater activity as rurality increases.

## 1 Introduction

Delivering health services using live, two-way video technology (telehealth) and providing financial assistance for patients who need to attend healthcare that is unavailable to them locally are two strategies commonly used to reduce healthcare access inequity. Although these programs have been established to address the same challenge, they take vastly different approaches and for this reason they are often administered without consultation with one another. Comparing how TH and PTSS programs are accessed by patients may help to identify where gaps exist in program delivery and improve patient choice for those located in regional and remote Queensland (Australia).

Queensland's telehealth (TH) program has been operating for several decades, sustained by $30.9 million in recurrent funding since the 2013-14 financial year from the state government [1]. This funding has been used to establish a workforce to coordinate and support telehealth activity and an incentive program encouraging clinicians to provide virtual services where it is clinically appropriate to do so [1,2]. Currently, between 3 and 4% of Queensland's outpatient activity is delivered by TH either to patients located at regional health centres or directly into their homes and is available to patients living in rural or metropolitan settings [3]. The clinical and economic impacts to patients are regularly evaluated [[4], [5], [6], [7], [8]].

The Patient Travel Subsidy Scheme (PTSS) in Queensland spent $84.25 million budget in the 2022-23 financial year, assisting patients to access public or private specialist medical services that are unavailable locally [9]. PTSS acts as a subsidy, rather than a reimbursement and is not designed to cover all costs associated with accessing healthcare (i.e. the program offers patients $70 per night towards accommodation costs and 34 cents per kilometre if the journey is made by private vehicle). Subsidies are also available for journeys by air, bus, rail, or ferry [9]. The PTSS is also externally evaluated to ensure that principles of patient safety, reduction in access inequity and the efficient use of public resources are maintained [10]. Some limitations have been placed on accessing PTSS by residents of Queensland living in metropolitan regions due healthcare access issues being comparatively small [10].

## 2 Method

Data extracts from corporate and local systems containing PTSS financial records were analysed and a record of all activity for the 2022/23 financial year were compiled (n = 270,933). This record included a count of all PTSS claims made and the postcode from the claimant's residential address. Similarly, records for all outpatient appointments delivered by telehealth over the same period were extracted (n = 250,171). Standardised rates of usage for both PTSS and TH (per 1000) were calculated for each postcode using population data from the 2021 census [11]. Postcode data was also used to group usage data by HHS and ARIA. Correlation and regression analysis was performed on rates of program usage by postcode, HHS and ARIA [12,13].

## 3 Results

The correlation analysis conducted on PTSS and TH activity revealed a statistically significant, moderate positive correlation (r = 0.449, p < 0.01). The Pearson correlation coefficient of 0.449 indicates that as PTSS increases, there is a tendency for TH to also increase in each postcode. Regression analysis with PTSS activity as a predictor revealed the model was statistically significant (F = 108.651, p < 0.001), and the coefficient for TH was 0.837 (p < 0.001), suggesting a positive relationship. An increase in units of telehealth activity led to an increase in PTSS activity by up to 0.837 units. TH (coefficient = 0.650, p < 0.001) and rurality (coefficient = 26.208, p = 0.686) also retained their significance.

## 4 Discussion

TH and PTSS are made available and accessed by the population of Queensland to reduce the effects of healthcare access inequity. The positive correlation between TH and PTSS activity identified in the analysis indicates that an increase in the rate of TH is a predictor of higher rates of PTSS with rates of both increasing with rurality. The TH program demonstrated an ability to facilitate similar levels of activity to PTSS at lower cost to the state health department.

Although each TH event represents an occasion where travel (and a potential PTSS claim) was avoided, a threshold in which there is sufficient TH activity to cause a reduced demand for PTSS was not observed in this study. This may be because such an effect is too sensitive to be observed using the whole-of-organisation scale of this study. More targeted evaluations may demonstrate, for example, that a newly established telehealth service can locally reduce travel subsidy need in specific geographical areas for specific clinic types.

An alternative explanation is that unmet, unexpressed demand for clinical services exists and making these available in ways that are more suitable to regional and remote populations increases demand. Although PTSS and TH are administered independently, they are complementary, and each program is essential to reduce healthcare access inequity in an economical manner. PTSS is needed where healthcare must be delivered in person such as procedures and examinations. The ability for TH to avoid patient travel for consultations (in particular, pre or post operative review appointments), offers benefits to both patients and the state health service. A valid and repeatable method of quickly identifying patients that would be suitable for TH appointments within the context of a busy outpatient department setting remains a challenge to many healthcare providers.

**Fig. 1:**
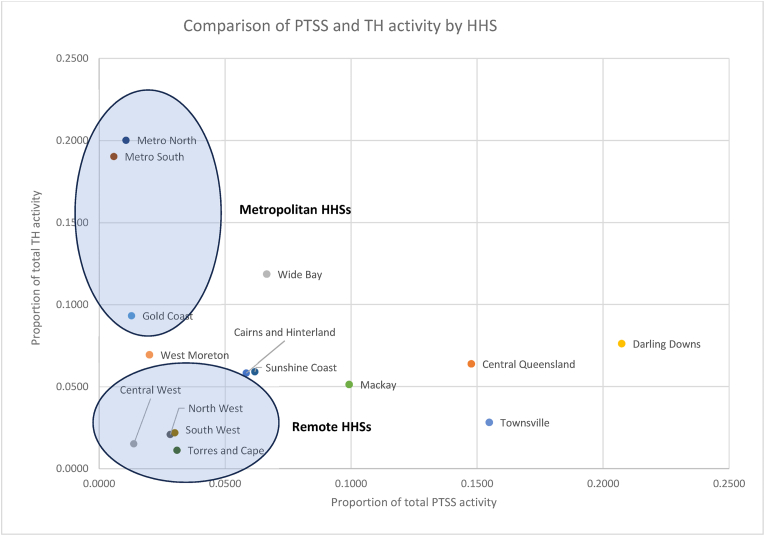
Scatterplot of HHSs by proportion of total statewide PTSS and TH activity.

**Fig. 2:**
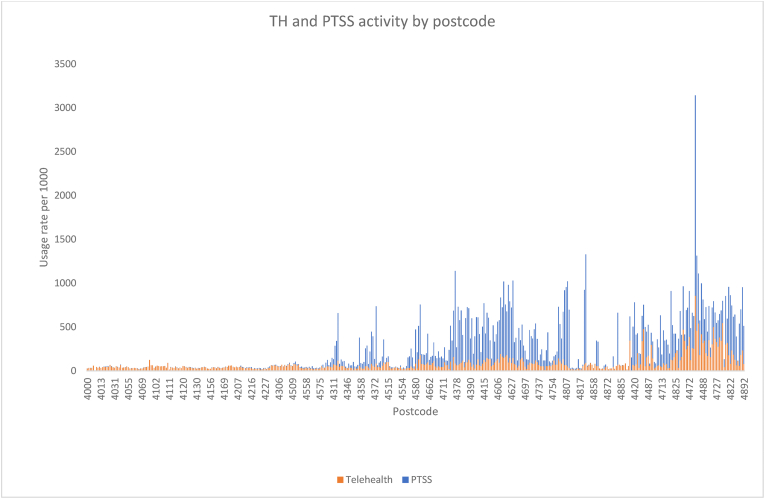
Proportion of TH and PTSS by Postcode.

Fig. 1 and Fig. 2 establish that metropolitan HHSs with large populations and sparsely populated remote HHSs both make relatively small contributions to the total PTSS activity. HHSs located in regional Queensland with a decentralised, moderately sized population account for the majority of PTSS activity in the state. Paying close attention to regions where TH activity is lower relative to PTSS may provide opportunities for expanding TH and in doing so continue to reduce healthcare access inequity in a cost-effective manner.

## Data availability statement

Data available on request from the authors.

## Declaration of competing interest

No relevant disclosures.

## References

[1] The University of Queensland (Qld) (2020).

[2] Edirippulige S., Armfield N.R., Greenup P., Bryett A. (2016). Telehealth coordinators in hospital based telehealth services: who are they and what do they do?. J. Telemed. Telecare.

[3] Greenup E.P., Best D. (2023). Comparison of patient responses to telehealth satisfaction surveys in rural and urban populations in Queensland. Aust. Health Rev..

[4] Queensland Health (Qld) (2021). http://www.health.qld.gov.au/telehealth.

[5] Snoswell C.L., Smith A.C., Page M., Scuffham P., Caffery L.J. (2022). Quantifying the societal benefits from telehealth: productivity and reduced travel. Value.Health Reg. Issue.

[6] Snoswell C.L., Caffery L.J., Haydon H.M., Wickramasinghe S.I., Crumblin K., Smith A.C. (2019). A cost-consequence analysis comparing patient travel, outreach and telehealth clinic models for a specialist diabetes service to Indigenous people in Queensland. J. Telemed. Telecare.

[7] Cottrell M., Judd P., Comans T., Easton P., Chang A.T. (2019). Comparing fly-in fly-out and telehealth models for delivering advanced-practice physiotherapy services in regional Queensland: an audit of outcomes and costs. J. Telemed. Telecare.

[8] Snoswell C.L., North J.B., Caffery L.J. (2020). Economic advantages of telehealth and virtual health practitioners: return on investment analysis. JMIR Perioperative Med..

[9] The Queensland Cabinet and Ministerial Directory (Qld) (2023). https://statements.qld.gov.au/statements/98762.

[10] The Queensland Ombudsman (Qld) (2017). https://www.ombudsman.qld.gov.au/ArticleDocuments/495/PTSS20report20final.pdf.aspx.

[11] Australian Bureau of Statistics (2021). https://www.abs.gov.au/census/find-census-data/datapacks?release=2021&product=GCP&geography=POA&header=S.

[12] Queensland Health (Qld) (2023). https://www.health.qld.gov.au/system-governance/health-system/hhs.

[13] Australian Bureau of Statistics (2023). Remoteness areas. https://www.abs.gov.au/statistics/standards/australian-statistical-geography-standard-asgs-edition-3/jul2021-jun2026/remoteness-structure/remoteness-areas.

